# Randomized trial of amoxicillin vs. placebo for pneumonia in Pakistan: A double blind community-based randomized trial of amoxicillin versus placebo for fast breathing pneumonia in children aged 2-59 months in Karachi, Pakistan (RETAPP)

**DOI:** 10.1056/NEJMoa1911998

**Published:** 2020-07-02

**Authors:** Fyezah Jehan, Muhammad Imran Nisar, Salima Kerai, Benazir Balouch, Nick Brown, Najeeb Rahman, Arjumand Rizvi, Yasir Shafiq, Anita K M Zaidi

**Affiliations:** 1 Epidemiology and Biostatistics, Associate Professor Pediatrics and Pediatric Infectious Diseases, Aga Khan University, Pakistan, fyezah.jehan@aku.edu; 2 Epidemiology and Biostatistics, Assistant Professor, Aga Khan University, Pakistan, imran.nisar@aku.edu; 3 Epidemiology and Biostatistics, Research Specialist, Aga Khan University, Pakistan, Salima.kerai@aku.edu; 4 Research Specialist, Aga Khan University, Pakistan, Benazir.balouch@aku.edu; 5 Consultant Paediatrician, International Centre for Maternal and Child Health, Uppsala University, Uppsala, Sweden. Department of Paediatrics and Child Health, Aga Khan University, Karachi, Pakistan. Department of Child Health, Länssjukhuset Gävleborg, Sweden, nick.brown@kbh.uu.se; 6 Research Coordinator, Aga Khan University, Pakistan, najeeb.rahman@aku.edu; 7 Research Specialist, Aga Khan University, Pakistan, arjumand.rizvi@aku.edu; 8 Health Policy Management, Aga Khan University, Pakistan, Yasir.shafiq@aku.edu; 9 SM Microbiology and Infectious Diseases, Professor, Division of Women and Child Health, Aga Khan University, Pakistan, anita.zaidi@aku.edu

## Abstract

**BACKGROUND:**

World Health Organization (WHO) recommends oral amoxicillin in fast breathing pneumonia while recent trial evidence indicates that non-treatment might be non-inferior.

**METHODS:**

A double blind randomized parallel placebo-controlled non-inferiority trial was conducted in slums of Karachi, Pakistan. Children 2-59 months at primary health care centres fulfilling WHO criteria for fast-breathing pneumonia without danger signs were randomized to three days of placebo (test treatment) or amoxicillin using WHO weight bands. Primary outcome was cumulative treatment failure from randomization to completion of 3 days of treatment. A priori non-inferiority margin was set based on treatment failure of 3.5% in amoxicillin and 1.75% in placebo arm (NI margin of 1.75%).

**RESULTS:**

Between November 9 2014 and November 30 2017, 4002 children were randomized; placebo (n=1999) and amoxicillin (n=2,003). Per protocol failure rates for placebo group were 4.9 % (995/ 1927) and amoxicillin group were 2.6 %( 51/1929); difference 2.23 %( Upper bound of 95 % CI 3.24; P Value <0.001), also similar by intention to treat analysis. Fever, anaemia and wheeze predicted treatment failure. Number-Needed-to-Treat to prevent each failure was 44 (95 % CI 31-80). Mortality rates were 0.05% with one death per group. Relapse rates were 2.2 % and 3.1 % in the placebo and standard arms respectively.

**CONCLUSIONS:**

Non-inferiority was not established for placebo over amoxicillin in fast breathing pneumonia as difference in treatment failure was 2.3%, above the pre-specified margin of 1.75%. Low failure rates in placebo and high number needed to treat suggests that implications may be context specific.

**Trial Registration Number:** ClinicalTrials.gov number NCT02372461

## BACKGROUND

Pneumonia or acute lower respiratory tract infection (ALRI) is the leading cause of child morbidity and mortality in children under 5 years, causing 7·4 deaths per 1000 live births (CI 6·7–8·8) in 2015 (1). It is strongly associated with under nutrition and poverty, therefore, disproportionately affecting children living in impoverished and low and middle income countries (LMICs) (2-4). However, the epidemiology is changing rapidly partly as a result of incorporation of vaccination against major pathogens causing mortality in the pre-immunization era, *Haemophilus Influenza type B* and *Streptococcus Pneumoniae* (4, 5). Viral pathogens such as Respiratory Syncytial Virus, Metapneumovirus, rhinovirus and adenoviruses for which antibiotics are not indicated now cause the majority of ALRI (6, 7). Overuse of first line antibiotics inevitably results in the selection of beta-lactamase producing species many of which are now resistant to even third generation cephalosporin and carbapenem antibiotics (8, 9). Additional potential adverse effects include potential detrimental effects on gut microbiota (10, 11) and enteric immune system (12, 13).

In the primary care setting, the diagnosis of pneumonia is syndromal, based on a constellation of signs including age adjusted respiratory rate, fever, chest wall indrawing and symptoms such as poor feeding, cough and lethargy. In the absence of sophisticated diagnostics in low resource settings, the World Health Organization (WHO) recommends case management of children with common illnesses in community under integrated management of childhood illness (IMCI) algorithm (14).

Treatment allocation is made according to the severity of illness which is based on clinical criteria made by observation. After 2014, severity is classified into three categories: no pneumonia, mild pneumonia (fast breathing pneumonia OR chest indrawing) and severe pneumonia (with additional danger signs). Fast breathing pneumonia and chest indrawing pneumonia without a danger sign are felt appropriate for primary health care and home management with oral antibiotics while severe pneumonia requires secondary center referral, monitoring and parenteral antibiotic use.

The broad recommendation for children with ‘fast breathing pneumonia without danger signs’ is based on the assumption that a proportion of children in the most resource limited settings will not have the means to re-consult should the picture change. However, evidence for the guidance is weak and infections are often viral and self-limiting. This has generated substantial debate among experts.

In children with cough or difficulty breathing having age specific tachypnea (defined as respiratory rate of at least 50 breaths in an infant 2-11 months or at least 40 breaths or more in child 12-59 months) classified as ‘fast breathing’ pneumonia’, IMCI recommends the use of oral antibiotic (amoxicillin 50 mg/kg per day in two divided doses) for three days in areas of low HIV prevalence (14, 15).

The IMCI case management reliance on tachypnea means that it is a sensitive but non-specific tool and, therefore, has the potential to misclassify pneumonia leading to over-prescription of antibiotics. Research have shown that in most cases, children with cough and tachypnea only have mild upper respiratory tract infections instead of pneumonia when examined by physician or against chest radiograph (16-18).

In the setting of changing epidemiology where immunization has reduced the incidence of bacterial infection, there lies an equipoise regarding use of antibiotics for management of fast breathing pneumonia in children in community setting. In order to address this gap in knowledge, we conducted a knowledge, we conducted a knowledge, we conducted a

## METHODS

### TRIAL OVERSIGHT

This was an investigator initiated double blind randomized controlled non-inferiority trial at four Primary Health Care Centers (PHCs) run by the Department of Pediatrics and Child Health, Aga Khan University. The trial was overseen by a Technical Steering Committee (TSC) and a Data Safety and Monitoring Board (DSMB) (registered at ClinicalTrials.gov - NCT02372461). Initial version of protocol is published at https://www.ncbi.nlm.nih.gov/pmc/articles/PMC4710982/ and subsequent versions are available at NEJM. Ethics approval was given by Ethics Review Committee of Aga Khan University Pakistan with additional endorsement from the Faculty of Medicine Ethics Committee at Southampton University United Kingdom. All parents or legal guardians provided written, informed consent obtained by a non-study physician. The trial was performed in accordance with the principles of the Declaration of Helsinki. The authors assume responsibility for the accuracy and completeness of the data and analyses, as well as for the fidelity of the trial and this report to the protocol.

### PATIENTS

Participants who self-referred to primary health care centers (PHCs) in contiguously located peri urban sites in Karachi, Pakistan were recruited after being triaged for cough or difficulty breathing. We recruited children 2-59 months old who had World Health Organization (WHO) defined fast breathing pneumonia. A child met inclusion criteria if s/he presented with a respiratory rate of ≥ 50 breaths per minute (2-11 months) or ≥ 40 breaths per minute (12-59 months). A trained lady health worker and a physician assessed respiratory rate independently and a child was labeled as fast-breather if there was agreement. Wheeze was assessed through auscultation by study physician, all children with wheeze received up to three doses of inhaled bronchodilator according to WHO-IMCI guidelines for categorization of fast breathing with wheeze. After each bronchodilator use, respiratory rate was recounted and child was re-categorized for fast breathing. Only children having persistent fast breathing after a maximum of 3 inhalations were considered for enrolment irrespective of wheeze. This was done to remain in alignment with IMCI guidelines. Children were excluded if there was associated lower chest indrawing (a criterion for severe pneumonia in the guideline before 2014), any danger signs, and use of antibiotics in last 48 hours, hospitalization in last two weeks, pedal edema, known tuberculosis, asthma or other severe illness. Participants were also excluded if they were out of the catchment area enrolled in another study, or previously enrolled within the last six months. Children not enrolled in the last six months could become eligible for enrolment; this change in definition of previously enrolled was made based on efficiency and to increase the pace of recruitment. Six months’ time period was considered a sufficient washout period after discussion with the TSC and the DSMB.

### TRIAL PROCEDURES

Children were randomly allocated (1:1) to groups receiving either syrup Amoxicillin in two divided doses for three days, using IMCI recommended weight bands, or to placebo. Computer generated block randomization lists (4, 6, 8 and 10) stratified by age category (2-11 months and 12-59 months) were made by the AKU Clinical Trials Unit (CTU). The randomization lists containing the treatment allocation code were used by the CTU pharmacist to create labels pasted on white, sealed bottles containing amoxicillin or placebo. Both amoxicillin and placebo were reconstituted by the CTU pharmacist and stored at 4-8 degrees centigrade throughout use. Children were assigned a randomization identification number by physician blinded to the allocation code. This number was pasted on case files. Drug dispensing was undertaken by separate study staff in a segregated clinic area. Physicians who assessed outcomes were also masked to the drug dispensing. Physicians who were randomizing and assessing outcomes, participating parents as well as all trial study staff were blinded to the treatment allocation until the end of analysis of the study.

Each child’s baseline information was obtained regarding socio-demographics, air quality, birth, breastfeeding and immunization. Clinical history, examination and anthropometry were done. All doses were administered, recorded and observed by study staff at PHC in morning or during home visit in evening. Dose was repeated in case of vomiting within 15 minutes. Treatment failure was assessed using defined criteria on days 0, 1, 2 and 3 of randomization in morning by physician and in evening by community health worker. At each visit, history regarding other treatment, hospitalization, serious and non-serious adverse event was recorded. Relapse was assessed up to day 14 through follow up visits scheduled at days 5 and 14. Visits at day 8 and 12 were added to follow up schedule on DSMB recommendation after a death occurred in the trial. Children who missed their morning visits were visited at home by the physician. All children were assessed for vital status on day 21. Any deterioration detected either by a CHW during home visit (including borderline oxygen saturation defined as ≤92%), or by family at any time was promptly reported on a hotline number to a 24/7 on-call physician with facilitated referral to a tertiary care hospital if required. Children missing ≥ 2 doses including any of the first four doses were considered ineligible for per protocol analysis.

### OUTCOMES

Primary outcome was cumulative treatment failure (TF) from randomization until completion of the 3 day course of study drug. TF was indicated by using any of the following: death, WHO defined danger sign, onset of lower chest indrawing, hospitalization for any reason and change in study drug by study physician due to new onset comorbid infection or for serious non-fatal antibiotic associated adverse event. The null hypothesis was of inferiority of the placebo to amoxicillin: the alternative was of non-inferiority. Secondary outcome included relapse assessed between days 4-14 using the same criteria mentioned above. Treatment adherence was defined as follows: per protocol status (taking 5/6 or 6/6 doses by day 3) or adherence to the time of treatment failure if it occurred within 3 days with no other treatment. Adverse events were defined as: non severe (diarrhea, rash and mouth ulcer and severe adverse event) or severe (diarrhea requiring intravenous hydration, anaphylaxis, and organ failure, life threatening injury or death).

### STATISTICAL ANALYSIS

The initial sample size was based on a study by Hazir et al (18) specifying a TF of 5% with amoxicillin and a non-inferiority (NI) margin of 2.5% (50 % greater than the standard treatment). After interim analysis of 1,000 participants, the overall TF was found to be lower, 3.5%. Therefore sample size was revised to specify a more conservative NI margin of 1.75% with a presumed TF of 3.5% with amoxicillin. With this NI margin, a minimum of 3,778 children were required to complete treatment per-protocol to achieve 90% power at one sided alpha of 0.05. Considering a non-per protocol rate of 5% a minimum of 3,978 were, therefore, required.

All data were doubled entered using a customized SQL-based relational database management system with an audit trail. Interim analyses were performed using an O’Brien-Fleming approach for the purpose of safety by an independent statistician supervised by DSMB. A statistical and/or clinically meaningful difference in adverse events including death was sought between the two arms in a blinded manner, at a significance level of α= 0.01 for stopping trial.

A statistical analysis plan was created and approved by the DSMB in a face to face meeting in Istanbul attended by all DSMB and TSC members and observers from the granting agency before locking of the database and un-blinding of treatment arms.

For the analysis of primary outcome which was per-protocol, unadjusted proportions and risk difference of treatment failure (TF_placebo-_ TF_amoxicillin_) with one sided 95% CI was estimated as per standard for NI trials. NI margin of 1.75 was used and non-inferiority would be demonstrated if upper bound of the one sided 95% CI of difference lied below this. Both per-protocol and intention-to-treat analysis were done. The proportion of children and risk difference between groups experiencing non-serious and serious adverse events was estimated using the same one-sided 95 % CI as for the main outcome and P values were reported. Stratified analysis was done by age categories, fever and wheeze as pre-specified in the protocol and presented using point estimates and 95%CIs.

As additional analyses, we also identified overall predictors of treatment failure using a backward elimination logistic regression model. At the outset, univariate analysis was conducted to identify the effect of each predictor on outcome. Variables significant at a p-value of 0.2 or less at univariable level were considered for adjustment in multivariable model. Due to different cutoffs of respiratory rate (RR) by age RR was kept as continuous variable in the model. A p-value of 0.05 was considered significant.

We also assessed the impact of re-randomization on treatment failure using mixed effects logistic regression, to account for potential non-independence between observations from re-enrolled children.

Secondary outcomes (including relapse) were compared using raw incidence data as we had no a priori information on which to base inferiority margins. The number needed to treat to prevent a treatment failure was calculated. All analyses were done using STATA version 14.

## RESULTS

### CHARACTERSTICS OF THE PATIENTS

**Figure 1:**
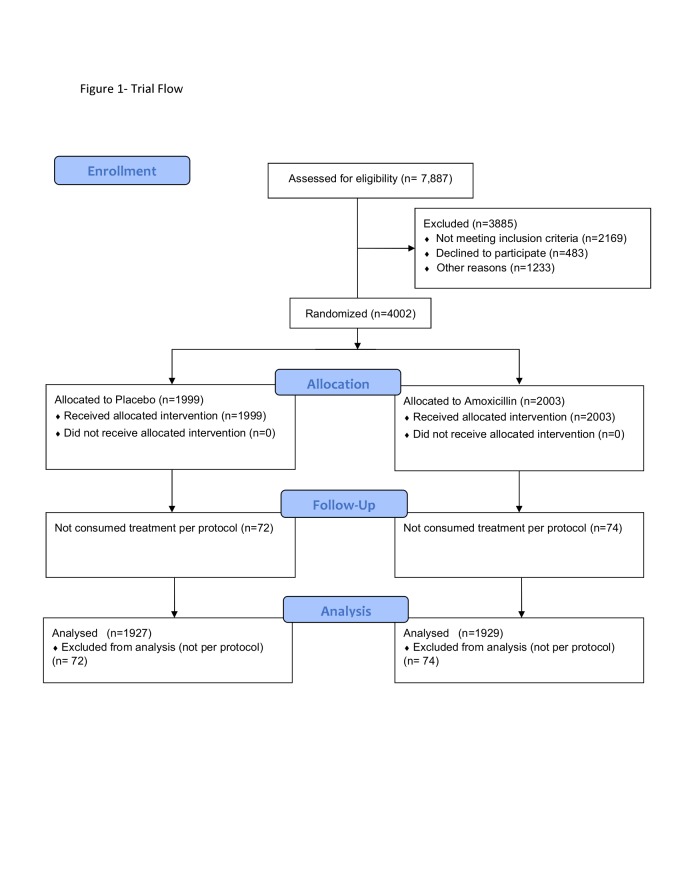
Trial Flow

From November 9 2014 to November 30 2017, 4,002 children were randomly assigned to receive amoxicillin (2,003) or placebo (1,999). Of these 1,929 (96.3 %) in amoxicillin arm and 1,927(96.4 %) in placebo arm were fully adherent and therefore available for per-protocol analysis (Figure 1). There was one protocol deviation that was not included in the analysis.

Table 1 provides the baseline characteristics of the study participants by treatment arm. Study groups were balanced with respect to important variables such as individual and household demographics, mean respiratory rates, mean oxygen saturation and other clinical characteristics. Of the children eligible for per protocol analysis (3,856) 53% were male and 46% were between 2-11 months of age.

**Table 1:** Baseline Characteristics of all patients in the 2 treatment groups (n=4002)

	Placebo	Amoxicillin
**Demographic Indicators**		
Sex		
Male	1079/1999(53.9%)	1024/2003 (51.1%)
Age, months		
Mean(SD)	16.5(13.9)	16.4(14.0)
Median(IQR)	13(5-25)	13(5-24)
Age categories in month		
2-11	919/1999(45.9%)	922/2003(46.0%)
12-59	1080/1999(54.0%)	1081/2003(53.9%)
Maternal years of schooling		
No education	1239/1999(62.0%)	1257/2003(62.8%)
1-5 years	282/1999(14.1%)	284/2003(14.2%)
6-10 years	416/1999(20.8%)	396/2003(19.8%)
Above 10 years	62/1999(3.1%)	66/2003(3.3%)
**Reported symptoms of**		
Diarrhea	154/1999(7.7%)	161/2003(8.0%)
Fever	1169/1999(58.5%)	1200/2003(59.9%)
Cough	1986/1999(99.4%)	1988/2003(99.3%)
Fast/difficult breathing	1300/1999(65.0%)	1288/2003(64.3%)
Chest in-drawing	29/1999(1.5%)	22/2003(1.1%)
URTI	767/1999(38.4%)	745/2003(37.2%)
Vomiting	29/1999(1.5%)	42/2003(2.1%)
Measles(within last 3 month)	11/1999(0.6%)	17/2003(0.9%)
**Current Breastfeeding by Age**		
2-5 month	557/579(96.2%)	548/573(95.6%)
6-11 month	332/340(97.7%)	331/348(95.1%)
12-24 month	512/536(95.5%)	524/553(94.8%)
**Immunization ^a^**		
Up to date immunization	953/1999(47.7%)	1009/2003(50.4%)
Up to date PCV and Pentavalent	1218/1999(60.9%)	1243/2003(62.1%)
**Physical Examination**		
Anemia	200/1999(10.0%)	206/2003(10.3%)
MUAC <11.5 cm ^b^	43/1420(3.0%)	45/1430(3.1%)
Stunting(HAZ<-2 SD)	917/1985(46.2%)	903/1989(45.4%)
Wasting(WHZ<-2SD)	349/1973(17.7%)	371/1988(18.7%)
Under-weight(WAZ<-2SD)	823/1993(41.3%)	838/2001(41.9%)
Temperature >=37.5 °C	652/1999(32.6%)	653/2003(32.6%)
Child Respiratory rate, breaths/min		
Mean(SD)	46.1(5.1)	46.3(5.3)
Median(IQR)	44.5(42.5-48.0)	45.0(42.5-48.5)
40-49	877/1080(81.2%)	864/1081(79.9%)
50-59	175/1080(16.2%)	191/1081(17.7%)
60-69	26/1080(2.4%)	22/1081(2.0%)
>=70	2/1080(0.2%)	4/1081(0.4%)
Infant Respiratory rate, breaths/min		
Mean(SD)	55.6(4.8)	55.2(4.7)
Median(IQR)	54.5(52.0-57.5)	54.0(52.0-57.0)
50-59	768/919(83.6%)	796/922(86.3%)
60-69	138/919(15.0%)	110/922(11.9%)
70-79	12/919(1.3%)	13/922(1.4%)
>=80	1/919(0.1%)	3/922(0.3%)
Oxygen Saturation, %		
90-92	251/1999(12.6%)	257/2003(12.8%)
93-95	552/1999(27.6%)	525/2003(26.2%)
>95	1196/1999(59.8%)	1221/2003(61.0%)
Wheeze	150/1999(7.5%)	134/2003(6.7%)
**Household demographics**		
HH with improved drinking water ^c^	1829/1999(91.5%)	1821/2003(90.9%)
HH with improved sanitation facilities ^d^	1839/1999(92.0%)	1829/2003(91.3%)
HH indoor air quality ^e^		
Good	1047/1999(52.4%)	1092/2003(54.5%)
Moderate	354/1999(17.7%)	332/2003(16.6%)
Poor	598/1999(29.9%)	579/2003(28.9%)

Data reported as n/N(%) for categorical and mean(Standard deviation-SD) & median(Interquartile Range-IQR) for continuous variables

a Children received age appropriate vaccines doses as per the standard immunization schedule

b MUAC (Mid Upper Arm Circumference) not applicable for children under 6 month of age

c HH (Household) with Improved drinking water includes piped water, tube well and bottled water

d HH with Improved sanitation facilities included septic tanks and pit latrine

e Indoor air quality index were created from 6 air quality variables includes HH have proper ventilation, smoking inside house, source of fuel wood ,charcoal or animal dung, cooking place , stove type, presence of child near cooking area using Principal Component Analysis. Quartiles were created from PCA score to group sampled population into three categories. A binary variable further created combining lower two quartiles reflecting inadequate air quality.

WAZ-Weight for age Z score, HAZ-Height for age Z score, WHZ-Weight for height Z score

### PRIMARY OUTCOME RESULTS

Treatment failures were 51(2.6 %) with amoxicillin and 95 (4.9 %) with placebo including one death in each arm. Relapses were 58 (3.1 %) in the amoxicillin arm and 40 (2.2 %) in placebo. There were 43 (2.3 %) and 63 (3.3 %) adverse events respectively. Treatment failure rates by day 3 (and including death at any time) showed an unadjusted difference of 2.3 % between the groups, with an upper 95 % CI of 3.7 % (favoring amoxicillin). This was above the non-inferiority margin of 1.75% (table 2) which suggested that we failed to reject the null hypothesis of inferiority of placebo. The results were similar when analysis was repeated using intention to treat.

**Table 2:** Outcomes by Day 3 and Day 14

Outcome	Placebo	Amoxicillin	Difference(95% CI)	P
	n/N (%)	n/N (%)		Value
**Day 3**				
PP	95/1927(4.9%)	51/1929(2.6%)	2.29(0.91,3.66)	<0.001
ITT	96/1999(4.8%)	51/2003(2.6%)	2.26(0.93,3.59)	<0.001
				
Adverse events				
PP	63/1927(3.3%)	43/1929(2.2%)	1.04(-0.14,2.22)	0.04
ITT	66/1999(3.3%)	46/2003(2.3%)	1.01(-0.16,2.17)	0.05
				
**Day 4-14**				
Relapse				
PP	40/1833(2.2%)	58/1878(3.1%)	-0.91(-2.08,0.27)	0.08
ITT	41/1904(2.2%)	58/1952(3.0%)	-0.82(-1.96,0.32)	0.11
				
Adverse events				
PP	39/1833(2.1%)	59/1878(3.1%)	-1.01(-2.19,0.16)	0.05
ITT	40/1904(2.1%)	59/1952(3.0%)	-0.92(-2.06,0.22)	0.07

Abbreviations: CI, confidence interval; ITT ,intention to treat ; PP, per protocol Data reported as n/N (%) Outcomes of day 4-14 calculated from those with treatment success (at days 0-3)

### PREDICTORS OF TREATMENT FAILURE

The comparison of baseline characteristics of children in the treatment failure and treatment success groups is shown in Table 3. Univariable analyses showed that treatment failure was statistically associated with history of diarrhea OR1.8 (95% CI 1.1-3.0); fever OR2.2 (95% CI 1.5-3.2), anemia OR1.7 (95% CI 1.1-2.7), body temperature ≥37.5 0C OR 2.2 (95% CI: 1.6-3.1) and wheeze OR 2.0 (95% CI 1.2-3.3). Using respiratory rate of < 60 breaths per minute as a reference in infants aged 2-11 months, there was a clear gradient in TF, 60-69 breaths per minute OR 1.9 (95% CI 1.1-3.5) , 70-79 breaths per minute OR 4.3 (95% CI 1.2-14.9), > 80 breaths per minute OR 9.6 (95% CI 1-93.5) compared to 50-59 breaths per minute.

**Table 3:** Comparison of no treatment failure and treatment failure (by Day 3) by Baseline Characteristics of Patients Completed per Protocol Treatment (n=3856)

	No treatment failure	Treatment failure	OR (95% CI)	P value
**Treatment**				
Amoxicillin	1878/3710(50.6)	51/146(34.9)	Reference	
Placebo	1832/3710(49.4)	95/146(65.1)	1.9(1.4-2.7)	<0.001
**Demographic Indicators**				
Sex				
Male	1948/3710(52.5)	82/146(56.2)	1.2 (0.8-1.6)	0.39
Age categories in month				
2-11	1707/3710(46.0)	70/146(48.0)	Reference	
12-59	2003/3710(54.0)	76/146(52.1)	1.1 (0.8-1.5)	0.65
Maternal years of schooling				
No education	2329/3710(62.8)	88/146(60.3)	0.9 (0.3-2.2)	0.75
1-5 years	521/3710(14.0)	22/146(15.1)	1.0 (0.4-2.6)	0.94
6-10 years	746/3710(20.1)	31/146(21.2)	0.9 (0.4-2.5)	0.91
Above 10 years	114/3710(3.1)	5/146(3.4)	Reference	
**Reported symptoms of**				
Diarrhea	280/3710(7.6)	19/146(13.0)	1.8 (1.1-3.0)	0.02
Fever	2164/3710(58.3)	110/146(75.3)	2.2 (1.5-3.2)	<0.001
Cough	3683/3710(99.3)	145/146(99.3)	1.1 (0.1-7.9)	0.95
Fast/difficult breathing	2387/3710(64.3)	104/146(71.2)	1.4 (1.0-2.0)	0.09
Chest in-drawing	47/3710(1.3)	2/146(1.4)	1.1 (0.3-4.5)	0.91
URTI	1397/3710(37.7)	55/146(37.7)	1.0 (0.7-1.4)	0.99
Vomiting	63/3710(1.7)	3/146(2.1)	1.2 (0.4-3.9)	0.75
Measles(within last 3 month)	26/3710(0.7)	1/146(0.7)	1.0 (0.1-7.3)	0.98
**Breastfeeding with age categories**				
2-5 month	1023/1069(95.7)	44/44(100.0)	--	--
6-11 month	616/637(96.7)	22/26(84.6)	0.2 (0.1-0.6)	0.004
12-24 month	956/1007(94.9)	44/45(97.8)	2.3 (0.3-	0.40
**Immunization ^a^**				
Up to date immunization	1838/3710(49.5)	61/146(41.8)	0.7 (0.5-1.0)	0.07
Up to date PCV and Pentavalent	2293/3710(61.8)	89/146(61.0)	1.0 (0.7-1.4)	0.84
**Physical Examination**				
Anemia	369/3710(10.0)	23/146(15.8)	1.7 (1.1-2.7)	0.02
MUAC <11.5 cm ^b^	80/2641(3.0)	4/102(3.9)	1.3 (0.5-3.6)	0.61
Stunting(HAZ<-2 SD)	1687/3684(45.8)	71/144(49.3)	1.2 (0.8-1.6)	0.41
Wasting(WHZ<-2SD)	664/3673(18.1)	30/142(21.1)	1.2 (0.8-1.8)	0.36
Under-weight(WAZ<-2SD)	1543/3703(41.7)	67/145(46.2)	1.2 (0.9-1.7)	0.28
Temperature ≥37.5 °C	1175/3710(31.7)	74/146(50.7)	2.2 (1.6-3.1)	<0.001
RR child				
40-49	1620/2003(80.9)	53/76(69.7)	Ref.	
50-59	334/2003(16.7)	19/76(25.0)	1.7 (1.0-3.0)	0.04
60-69	44/2003(2.2)	3/76(4.0)	2.1 (0.6-6.9)	0.23
≥70	5/2003(0.3)	1/76(1.3)	6.1 (0.7-	0.10
RR Infant				
50-59	1463/1707(85.7)	51/70(72.9)	Reference	
60-69	221/1707(13.0)	15/70(21.4)	1.9 (1.1-3.5)	0.04
70-79	20/1707(1.2)	3/70(4.3)	4.3 (1.2-	0.02
≥80	3/1707(0.2)	1/70(1.4)	9.6 (1.0-	0.05
Oxygen Saturation				
90-92	467/3710(12.6)	23/146(15.8)	Reference	
93-95	990/3710(26.7)	44/146(30.1)	0.9 (0.5-1.5)	0.69
>95	2253/3710(60.7)	79/146(54.1)	0.7 (0.4-1.1)	0.16
Wheeze	261/3710(7.0)	19/146(13.0)	2.0 (1.2-3.3)	0.007
**Household demographics**				
HH with Improved drinking water^c^	3388/3710(91.3)	130/146(89.0)	0.8 (0.5-1.3)	0.34
HH with Improved sanitation^d^	3406/3710(91.8)	137/146(93.8)	1.4 (0.7-2.7)	0.38
**HH indoor air quality^e^**				
Adequate	1991/3710(53.7)	61/146(41.8)	Reference	
Inadequate	1719/3710(46.3)	85/146(58.2)	1.6 (1.2-2.2)	0.005

Data reported as n/N(%)

a Children received age appropriate vaccines doses as per the standard immunization schedule

b MUAC (Mid Upper Arm Circumference) not applicable for children under 6 month of age

c HH (Household) with Improved drinking water includes piped water, tube well and bottled water

d HH with Improved sanitation facilities included septic tanks and pit latrine

e Indoor air quality index were created from 6 air quality variables includes HH have proper ventilation, smoking inside house, source of fuel wood ,charcoal or animal dung, cooking place , stove type, presence of child near cooking area using Principal Component Analysis. Quartiles were created from PCA score to group sampled population into three categories. A binary variable further created combining lower two quartiles reflecting inadequate air quality.

WAZ-Weight for age Z score, HAZ-Height for age Z score, WHZ-Weight for height Z score

Adjusted for all factors (Table 4) treatment with amoxicillin was associated with a significant reduction in odds of treatment failure OR 0.51 (95% CI 0.36-0.73). Other independent predictors of treatment failure included: respiratory rate OR 1.05 (95% CI 1.03-1.08); wheezing OR 1.79 (95%CI 1.08-2.98); fever OR 1.59 (95% CI 1.03-2.46) and anemia OR 1.67 (95% CI 1.03-2.69) at presentation; history of fever OR 1.59 (95% CI 1.03-2.46) and diarrhea OR 1.66 (95% CI: 1.00-2.76) and inadequate indoor air quality OR 1.41 (95% CI 1.00-1.99).

**Table 4:** Multivariable analysis to compare no treatment failure with treatment failure (by Day 3) (n=3856)

	Adjusted Odds Ratio	95% CI	P-value
Treatment			
Amoxicillin	Reference		
Placebo	1.95	1.37-2.77*	<0.001
History of diarrhea	1.66	1.00-2.76	0.05
History of fever	1.59	1.03-2.46	0.04
Anemia at presentation	1.67	1.03-2.69	0.04
Temperature ≥37.5 °C	1.68	1.15-2.44	0.007
Wheeze at presentation	1.79	1.08-2.98	0.03
Respiratory rate^a^	1.05	1.03-1.08*	<0.001
HH indoor air quality			
Adequate	Reference		
Inadequate	1.41	1.00-1.99	0.05

a For an increase of respiratory rate by 1 breath per minute. HH, Household

To see the impact of re-enrolment, child level variability showed an intra-class correlation = 0, variance=0.0001, suggesting that episodes could be treated independently even when the same child was enrolled more than once. The effects of anemia OR 1.6 (95%CI 0.93-2.76), stunting OR 1.41 (95% CI 0.96-2.07) and wheeze OR 1.78 (95% CI 0.98-3.23) were of borderline significance in the mixed model. Reported fever OR 2.22 (95% CI 1.43-3.45) and documented fever OR 2.42 (95% CI 1.70-3.65) remained significant. The analysis results are presented in Supplementary table S1.

### SECONDARY OUTCOME RESULTS

Analysis of secondary outcomes showed no difference in rates of relapse; mean difference 1.01 (95% CI -0.16-2.19) and no difference in adverse events mean difference 1.01 (95% CI -0.16-2.19).

In subgroup analysis by age, presence of fever and wheeze, placebo was again not found to be non-inferior to amoxicillin. Difference in treatment failures in placebo minus amoxicillin was higher in the presence of fever 4.08 (upper 95% CI 6.68) and wheeze 7.01(upper 95% CI 12.67). (Table 5).

**Table 5:** Stratified sub-group analysis of outcomes (Per-protocol)

	N	Placebo (n/N) %	Amoxicillin (n/N) %	Difference (95% CI)
	**Treatment failure(0-3 day)**			
**Age**				
2 to <12 months	1,776	46/881(5.2%)	24/895(2.7%)	2.54(0.73,4.35)
12 to < 60 months	2,080	49/1046(4.7%)	27/1034(2.6%)	2.07(0.47,3.68)
**Fever(Temperature ≥37.5 °C)**				
Present	1,249	50/629(8%)	24/620(3.9%)	4.08(1.48,6.68)
Absent	2,607	45/1298(3.5%)	27/1309(2.1%)	1.4(0.15,2.66)
**Wheeze**				
Present	280	15/149(10.1%)	4/131(3.1%)	7.01(1.35,12.67)
Absent	3,576	80/1778(4.5%)	47/1798(2.6%)	1.89(0.67,3.1)
	**Adverse events (0-3 day)**			
**Age**				
2 to <12 months	1,776	30/881(3.4%)	24/895(2.7%)	0.72(-0.87,2.32)
12 to < 60 months	2,080	33/1046(3.2%)	19/1034(1.8%)	1.32(-0.02,2.66)
**Fever(Temperature >=37.5 °C)**				
Present	1,249	32/629(5.1%)	20/620(3.2%)	1.86(-0.35,4.07)
Absent	2,607	31/1298(2.4%)	23/1309(1.8%)	0.63(-0.46,1.73)
**Wheeze**				
Present	280	4/149(2.7%)	0/131(0%)	2.68(0.09,5.28)
Absent	3,576	59/1778(3.3%)	43/1798(2.4%)	0.93(-0.16,2.02)
	**Relapse (4-14 day)**			
**Age**				
2 to <12 months	1,707	21/836(2.5%)	34/871(3.9%)	-1.39(-3.06,0.28)
12 to < 60 months	2,004	19/997(1.9%)	24/1007(2.4%)	-0.48(-1.75,0.79)
**Fever(Temperature >=37.5 °C)**				
Present	1,175	13/579(2.3%)	21/596(3.5%)	-1.28(-3.19,0.63)
Absent	2,536	27/1254(2.2%)	37/1282(2.9%)	-0.73(-1.95,0.49)
**Wheeze**				
Present	261	6/134(4.5%)	6/127(4.7%)	-0.25(-5.33,4.84)
Absent	3,450	34/1699(2%)	52/1751(3%)	-0.97(-2.01,0.07)
	**Adverse events (4-14 day)**			
**Age**				
2 to <12 months	1,707	17/836(2%)	30/871(3.4%)	-1.41(-2.95,0.13)
12 to < 60 months	2,004	22/997(2.2%)	29/1007(2.9%)	-0.67(-2.05,0.7)
**Fever(Temperature >=37.5 °C)**				
Present	1,175	12/579(2.1%)	14/596(2.4%)	-0.28(-1.96,1.4)
Absent	2,536	27/1254(2.2%)	45/1282(3.5%)	-1.36(-2.65,-0.07)
**Wheeze**				
Present	261	0/134(0%)	2/127(1.6%)	-1.57(-3.74,0.59)
Absent	3,450	39/1699(2.3%)	57/1751(3.3%)	-0.96(-2.05,0.13)

In order to prevent one treatment failure, the number needed treat was 40 children with fast breathing pneumonia.

### DISCUSSION

In the RETAPP trial conducted in a low income, non HIV, low malaria endemic, primary health care setting, the overall rates of treatment failure were not the same in the placebo arm as compared to Amoxicillin. This included one death in the Amoxicillin arm on day 3 and one death in the placebo arm on day 13. There were more relapses in the amoxicillin arm between Days 4 to 14 however results did not achieve statistical significance.

In the study, 18.6% of children had moderate to severe wasting and over half of the children had received the third dose of pentavalent and pneumococcal vaccines. We included malnourished children and the results did not differ by this subgroup analysis. The results also did not differ when stratified by infants and older children.

There is a paucity of literature on withholding of antibiotics for this relatively benign and common condition in children from low middle income settings, the conclusions of which are inconsistent. Our findings, therefore, need to be contextualized.

In Hazir’s study in outpatient care in Pakistan, the day 3 failure rates were higher but comparable (7.2 % and 8.3% respectively indicating equivalence), but their population included far more wheezing children (50%) so the findings potentially are not generalizable (18). Awasthi et al, on the other hand, showed significant differences between outcomes in children unresponsive to bronchodilators with a normal chest X ray in an Indian ambulatory care setting with failure rates substantially higher (19.9 % and 24 %, difference 4.2 %, 95% CI 0.2 to 8.1 %) without amoxicillin (19). In this study, persistence of wheeze was used as a criterion for failure which again limits generalizability. Our trial included far fewer children with wheeze and none that showed a bronchodilator response, in other words were likely to have included a greater proportion of children with a primarily infectious cause of their fast breathing.

A recently published study by Ginsburg et al in Malawi, Africa made a similar comparison as RETAPP and was halted prematurely because placebo showed larger failure rates compared with amoxicillin (7% vs. 4%), however the study included fever as an additional criteria of treatment failure and did not include any deaths and was conducted in outpatient departments of hospitals (20).

What then are the resource and policy implications of these findings? The message appears to be that, on the basis of the current WHO categorization, existing recommendations are valid but there are a number of important riders. These include numbers needed to treat and economics, targeting subgroups and relapse.

The first is that the number of children with fast breathing pneumonia needed to treat to prevent one treatment failure was high. Since there was no difference in mortality or relapse in favor of amoxicillin, this figure demands that cost effectiveness evaluation is undertaken. The costs are broadly twofold. The first is the raw expenditure on antibiotics which as Zangh et al’s systematic review of costs in severe pneumonia suggested are considerable at both the individual and societal levels (21). The second, very real cost is that of exacerbating the already widespread levels of antibiotic resistance globally. Beta lactam resistance is at epidemic levels in parts of Asia (8, 9) and the only sure means of preventing further extension to cephalosporins and carbapenems is by stewardship. In an era in which the epidemiology of pneumonia has changed rapidly as a result of the success of the pneumococcal and Haemophilus Influenza B vaccination programs (4), the equation is a complex one and the observation that neither wasting or stunting, nor vaccination status predicted treatment failure lends weight to the hypothesis that most children had viral infections. There was however increased morbidity with clinically detected anemia.

The second issue relates to the criteria for pneumonia categorization. Though not powered for subgroup analyses, RETAPP raises several questions about targeted treatment. Temperature greater than or equal to 37.5°C, anemia and the presence of wheeze all significantly predicted TF. In the absence of these signs, allocation groups were comparable. Reported temperature, documented fever and wheeze at presentation have also been identified to be of prognostic importance in children with acute cough and respiratory infections in developed countries.(22).

This suggests that, although potentially more complex to manage in the primary care setting, that with a change in criteria for non-severity, screening by predictor variables might be more cost effective. One issue which is now unequivocal is that the roll out of oxygen saturation testing at presentation will enhance the process (23). Debate continues as to whether point of care biomarker testing will augment triage (24).

The third issue relates to the intriguing difference in relapse rates. There were fewer TFs but greater relapse rates (3 % vs. 2 %) in the antibiotic group. What does this mean biologically? One explanation might be that it represents a subgroup of children with resistant bacterial infection who were treated or suppressed and then relapsed. Could these be the same children who would have been the TFs in the placebo group who simply presented earlier? Though hard to prove, this seems possible and represents an interesting philosophical balance/’trade off’. resistant bacterial infection who were treated or suppressed and then relapsed. Could these be the same children who would have been the TFs in the placebo group who simply presented earlier? Though hard to prove, this seems possible and represents an interesting philosophical balance/’trade off’.

Strengths of the study include the high adherence, directly observed treatment, low attrition, assiduous reporting and investigation of adverse events and per protocol analyses that are necessary for internal validity of non-inferiority studies.

Important limitations of the trial include those for generalizability because of the strict exclusion criteria and close follow-up beyond the normal for this population. The study area has high rates of health utilization for respiratory infections(25) and thus results may not be generalizable to settings where presentation is delayed and upon worsening of signs and symptoms, however this would be offset if such children would have been triaged to a higher level of care in that setting. Absence of radiology was a limitation, but this was a deliberate part of the design in order that the study replicated a ‘real life’ situation. Amoxicillin was prescribed using standard WHO recommended weight bands, which resulted in some children getting lower and some higher doses of amoxicillin. However none of the children received a dose less than 50mg/kg/day, the lower limit of high dose amoxicillin.

In conclusion we did not reject the null hypothesis of inferiority of placebo against amoxicillin in urban preschool Pakistani children with WHO-defined fast breathing pneumonia. Given that there is a low event rate with high number of self-resolving cases, there are implications for re-addressing respiratory rate cutoffs and identifying those at risk of treatment failure. A concerted effort by World Health Organization and partners is underway to use RETAPP and other such datasets to identify sub-populations who are at greater risk of failing treatment when not receiving antibiotics, in order to better specify children who would benefit most with antibiotics. Given that the margin of non-inferiority was small, adequately powered future trials may be required to further refine definition and classification of and treatment algorithms for fast breathing pneumonia.

### Sources of Support

This study was jointly funded by the MRC-Wellcome-DFID through the Joint Global Health Trials (grant MR/L004283/1) and Bill and Melinda Gates Foundation (OPP1158281). Fyezah Jehan and Muhammad Imran Nisar received training support from the Fogarty International Center, National Institute of Health (grant D43TW007585). The funders or the providers of study drugs had no role in the design, implementation or interpretation of the study. The corresponding author and statistician had full access to the data and the final responsibility to submit lay on the corresponding author. Fyezah Jehan and Imran Nisar wrote the first draft of the manuscript with considerable input from Nick Brown, Salima Kerai and Anita Zaidi. All authors reviewed and approved the final draft.
