# Supplementary material for: Randomized trial of amoxicillin vs. placebo for pneumonia in Pakistan: A double blind community-based randomized trial of amoxicillin versus placebo for fast breathing pneumonia in children aged 2-59 months in Karachi, Pakistan (RETAPP)
Source: N Engl J Med. Author manuscript; Available in PMC 2020 Jul 2. (PMC7244232; doi:10.1056/NEJMoa1911998)
Supplement: Supplementary file 1 [file NEJM-2020-1911998-s1.pdf]

Supplementary materials

Contents

1.1 Acknowledgments 2

1.2 Deaths 3

1.3 Supplementary Table 6

Table S1: Comparison of No treatment Failure and Treatment failure (by Day 3) by Baseline Characteristics of Patients Completed per Protocol Treatment (n=3856) using mixed model approach..6

1.4 Consort Checklist……………………………………………………………………………………………..8

1.1 Acknowledgments

The authors acknowledge the valuable contributions made by the RETAPP Study Team[a]

1	DR.BENAZIR	RESEARCH SUPERVISOR
2	SALIMA KERAI	RESEARCH SPECIALIST
4	SANA QAISER	RESEARCH ASSOCIATE
5	FAIZAN KHALID	RESEARCH ASSISTANT
6	DR.NARESH	RESEARCH ASSOCIATE
7	DR.KHIAR-UN-NISA	RESEARCH SPECILAIST
8	DR.BILQUIS MEHBOOB	RESEARCH ASSOCIATE
9	DR.DANIA MUSHTAQ	RESEARCH ASSOCIATE
11	DR.RAKHSHANDA FAHIM	RESEARCH ASSOCIATE
12	DR.IRUM AAMIR	RESEARCH ASSOCIATE
13	DR.SAIMA SIDDIQUI	RESEARCH ASSOCIATE
14	DR.RAKHSHANDA AYAZ	RESEARCH ASSOCIATE
16	HIRA KANWAL	Community Health Worker
17	YASMEEN ABDUL MUTALLIB	Community Health Worker
18	BASHIRA KHATOON	Community Health Worker
19	MAHNOOR M.IQBAL	Community Health Worker
20	AFSAR-UL-ISLAM	Community Health Worker
21	AISHA ABBAS	Community Health Worker
22	ANEESA MOHAMMAD	Community Health Worker
23	MARIUM AKRAM	Community Health Worker
24	TAHIRA ABBAS	Community Health Worker
25	SAIMA ABBAS	Community Health Worker
26	SHAHNAZ YAQOOB	Community Health Worker
28	SANA KHALID	Community Health Worker
29	SHAHIDA KANWAL	Community Health Worker
30	HINA REHMAN	Community Health Worker
31	SALMA TARIQ	Community Health Worker
32	ASIF LATIF	Community Health Worker
33	NAVEEN TAJAK	Community Health Worker
34	NOORIE ALI MOHAMMAD	Community Health Worker
35	YASMEEN	Community Health Worker
36	SHAISTA HONEY	Community Health Worker
37	ZEENAT AHMED	Community Health Worker
38	ULFAT IBRAHIM	Community Health Worker
39	BASHEER AHMED	PHLEBOTOMIST/CHW

The authors also thank the Clinical Trials Unit staff [b] including Dilshad Baig and Afshan (Pharmacist)

The authors also thank Dr Shamim Ahmed Qazi [c] for provision of technical guidance, oversight and advise during protocol implementation as Chair of the Trial Steering Committee.

We also thank Dr Patricia Hibberd [e] (Chair), Dr Chris Roberts and Dr Fauzia Qureshi [a] from the Data Safety and Monitoring Board for their guidance.

a. Department of Pediatrics and Child Health , Aga Khan University, Pakistan
b. Clinical Trials Unit, Aga Khan University, Pakistan
c. Department of Maternal Child and Adolescent Health, World Health Organization, Geneva
d. Department of Global Health, Boston University, USA.

1.2 Deaths

1. 4 months old male baby, weight 5.975, active alert, feeding well, no previous history of illness, 7th baby of the family delivered through normal vaginal delivery at home, cried immediately after birth, no significant family history of convulsions or any other illness, baby was exclusively breast feed.

   Enrolled with complaint of cough for 7 days and fever for 1 day. Respiratory rate was 64 breaths per minute (first reading), 64 breaths per minute (second reading), O2 saturation 99%, temperature 36.6 C, length 58.3/58.3cm, MUAC, 11.3/11.3 cm, vaccination incomplete (missed Pentavalent 3 and did not receive any dose of PCV).

   After randomisation, received 5ml of study drug at 1120 hours and dropped home after explaining and providing number of on call physician. It was also explained when to return in case of any emergency.

   Day 1_ November 6, 2016 at 0925 (Sunday) follow up conducted by CHW. On examination RR was 50/48/48, Temp 36C, O2 96%. Baby was well. Morning dose was given. Evening follow up and doses were also given by CHW in evening.

   Day 2 _ November 7, 2016 at 1150 hours baby was seen at health center and was examined by study physician. RR was 38/47/47; Temp 36C, O2 95%, morning dose was given. Evening follow up was conducted by CHW, baby was well and dose was given.

   Day 3_ November 8, 2016 at 1300 hours baby was examined at health center by study physician, RR was 43/35, Temp 36.9C, O2 99%. Baby was well.

   Day 5_ November 10, 2016 at 1345 hours. Baby was examined at health center by study physician, was well with no active complaints, feeding well, with RR 33/34, Temp 36.9C, O2 94%.

Day 14_November 19, 2016 As per schedule RETAPP CHW went to the baby's home today for 14 day follow up and was told by mother that patient had expired on 14-11-16 (Monday at 0400 hours in the hospital – National Institute of Child Health).

On Sunday, November 13, 2016 at 1100 hours baby developed high grade fever with convulsions, involving his right upper and lower limb with uprolling of the eyes, and frothing from mouth. He was taken to a local GP who referred him to Indus hospital by that GP without giving any medication. Patient became unconscious while on the way to Indus Hospital. He reached at 1300 to Indus Hospital. Doctors at Indus Hospital gave him some IV fluids, injectable drugs (not given any documented paper, they do not know the names of the medication) and oxygen therapy. Meanwhile mother was told that baby became stiff at that moment and was then referred to NICH at 1430 hours. At NICH patient was again given injectable drugs (names not known), IV fluids and then was intubated. The family was given ambu bag to ventilate the baby because of shortage of space in the Intensive Care Unit. He was kept in the emergency the whole time and expired at 0400 hours on November 14, 2016.

It was enquired why mother didn't contact on call physician and hospital number that was provided to her. She replied that she was confused to see the condition of the baby and forgot to call anyone. According to the mother they did not receive any discharge papers or treatment details.The death certificate mentioned cause of death is cardiopulmonary arrest secondary to pneumonia and sepsis.

2. 15 months old female child, active alert, feeding well, no previous history of illness, 2nd child of the mother delivered through normal vaginal delivery at some hospital, cried immediately after birth, no significant family history of convulsions or any other illness. Length 73.8/cm, MUAC, 12.4/12.4 cm, vaccination incomplete (missed Pentavalent 3 and pneumococcal 3 and measles 1 and 2).

   She was enrolled with complaints of cough for 2 days and fever for 1 day. Respiratory rate was 49 breaths per minute (first reading), 46 breaths per minute (second reading), O2 saturation 95%, temperature 40°C sponging done and Paracetamol given at the PHC, 2nd reading 38.6^{o}C, (physician gave him Syp paracetamol 4hrly for home). After randomization, received 5ml of study drug at 1120 hours and dropped home after explaining and providing number of on call physician and local collector Data collector of the study site. It was also explained when to return in case of any emergency.

   On Day 0, February 4, 2017 at 1811 hours evening follow up was conducted by CHW. On examination child had cough, RR was 44/46, Temp 36.7C, O2 92% in air. She was well according to parents. Evening dose, 5 ml of study drug was given by CHW the child vomited out and this was repeated which she tolerated. Father complained that the child had cough so requested CHW for nebulization, so the CHW nebulized the child with normal saline at the PHC, and then dropped them back at home, after explaining

to call in case of any emergency. Evening physician took update from the CHW who informed about the vomiting.

According to the father, after 2 hours of nebulization, the child developed high grade fever (not documented), and another bout of cough so parents took her to a local health care provider (GP) who prescribed injections ceftriaxone and amikacin along with oral ventolin syrup. After the injectable, according to the father, the child was better for some time but between 0100-0200 hours on February 15th 2017, he had two episodes of small watery loose stool. Within 5 to 10 minutes of the episode, the baby got stiff and unresponsive. The father felt like the child had already died but for confirmation, they took him to Sultan Hospital from where child was referred to Chinniot Hospital where the doctor declared death on arrival. Later, we asked the father why the on call physician was not contacted, to which they replied that they forgot to call. Based on the father's narrative, the child had died around 0200 hours at home.

1.3 Supplementary Table

Table S1: Comparison of No treatment Failure and Treatment failure (by Day 3) by Baseline Characteristics of Patients Completed per Protocol Treatment (n=3856) using mixed model approach

	No treatment failure	Treatment failure	OR (95% CI)
**Treatment**			
Amoxicillin	1878/3710(50.6)	51/146(34.9)	Ref.
Placebo	1832/3710(49.4)	95/146(65.1)	1.87(1.27,2.75)**
**Demographic Indicators**			
Sex			
Male	1948/3710(52.5)	82/146(56.2)	1.08(0.74,1.58)
Age categories in month			
2-11	1707/3710(46.0)	70/146(48.0)	Ref.
12-59	2003/3710(54.0)	76/146(52.1)	0.98(0.67,1.43)
Maternal years of schooling			
No education	2329/3710(62.8)	88/146(60.3)	1.28(0.31,5.33)
1-5 years	521/3710(14.0)	22/146(15.1)	1.20(0.27,5.37)
6-10 years	746/3710(20.1)	31/146(21.2)	1.32(0.30,5.80)
Above 10 years	114/3710(3.1)	5/146(3.4)	Ref.
**History of reported**			
Diarrhoea	280/3710(7.6)	19/146(13.0)	2.07(1.16,3.70)**
Fever	2164/3710(58.3)	110/146(75.3)	2.22(1.43,3.45)**
Cough	3683/3710(99.3)	145/146(99.3)	*
Fast/difficult breathing	2387/3710(64.3)	104/146(71.2)	1.34(0.89,2.02)
Chest in-drawing	47/3710(1.3)	2/146(1.4)	0.89(0.12,6.56)
URTI	1397/3710(37.7)	55/146(37.7)	1.23(0.84,1.81)
Vomiting	63/3710(1.7)	3/146(2.1)	0.62(0.08,4.56)
Measles(within last 3 month)	26/3710(0.7)	1/146(0.7)	*
**Breastfeeding with age categories**			
2-5 month	1023/1069(95.7)	44/44(100.0)	*
6-11 month	616/637(96.7)	22/26(84.6)	*
12-24 month	956/1007(94.9)	44/45(97.8)	*
**Immunization [b]**			
Upto date immunization	1838/3710(49.5)	61/146(41.8)	0.69(0.47,1.02)
Upto date PCV and Penta	2293/3710(61.8)	89/146(61.0)	0.97(0.65,1.42)
**Physical Examination**			
Anaemia	369/3710(10.0)	23/146(15.8)	1.60(0.93,2.76)
MUAC <11.5 cm [a]	80/2641(3.0)	4/102(3.9)	0.39(0.05,3.27)
Stunting(HAZ<-2 SD)	1687/3684(45.8)	71/144(49.3)	1.41(0.96,2.07)
Wasting(WHZ<-2SD)	664/3673(18.1)	30/142(21.1)	0.94(0.57,1.56)

	No treatment failure	Treatment failure	OR (95% CI)
Under-weight(WAZ<-2SD)	1543/3703(41.7)	67/145(46.2)	1.11(0.76,1.64)
Temparature >=37.5 °C	1175/3710(31.7)	74/146(50.7)	2.49(1.70,3.65)**
RR child			
40-49	1620/2003(80.9)	53/76(69.7)	Ref.
50-59	334/2003(16.7)	19/76(25.0)	2.29(1.01,5.21)**
60-69	44/2003(2.2)	3/76(4.0)	3.00(0.65,13.87)
>=70	5/2003(0.3)	1/76(1.3)	8.25(0.40,170.86)
RR Infant			
50-59	1463/1707(85.7)	51/70(72.9)	Ref.
60-69	221/1707(13.0)	15/70(21.4)	3.42(0.69,16.84)
70-79	20/1707(1.2)	3/70(4.3)	2.61(0.20,34.12)
>=80	3/1707(0.2)	1/70(1.4)	*
Oxygen Saturation			
90-92	467/3710(12.6)	23/146(15.8)	Ref.
93-95	990/3710(26.7)	44/146(30.1)	0.89(0.49,1.63)
>95	2253/3710(60.7)	79/146(54.1)	0.72(0.41,1.24)
Wheeze	261/3710(7.0)	19/146(13.0)	1.78(0.98,3.23)
**Household demographics**			
HH with improved drinking water	3388/3710(91.3)	130/146(89.0)	0.68(0.39,1.19)
HH with improved sanitation facilities	3406/3710(91.8)	137/146(93.8)	1.68(0.73,3.85)
HH indoor air quality [c]			
Good	1991/3710(53.7)	61/146(41.8)	Ref.
Moderate	625/3710(16.9)	38/146(26.0)	1.92(1.21,3.03)**
Poor	1094/3710(29.5)	47/146(32.2)	1.03(0.65,1.65)

** These variables were included in the final multivariate model

*Model not converged with very high and low indicator/outcome distribution

Consort Checklist

CONSORT Statement 2006- Checklist for Non-inferiority and Equivalence Trials

Items to include when reporting a non-inferiority or equivalence randomized trial

PAPER SECTION **And topic**	Item	**Descriptor**	**Reported on Page #**
TITLE & ABSTRACT	1	How participants were allocated to interventions (*e.g.*, "random allocation", "randomized", or "randomly assigned"), *specifying that the trial is a non-inferiority or equivalence trial.*	1, 3,4
INTRODUCTION Background	2	Scientific background and explanation of rationale, *including the rationale for using a non-inferiority or equivalence design.*	5,6,7
METHODS Participants	3	Eligibility criteria for participants *(detailing whether participants in the non-inferiority or equivalence trial are similar to those in any trial(s) that established efficacy of the reference treatment)* and the settings and locations where the data were collected.	7,8
Interventions	4	Precise details of the interventions intended for each group *detailing whether the reference treatment in the non-inferiority or equivalence trial is identical (or very similar) to that in any trial(s) that established efficacy,* and how and when they were actually administered.	8,9

Objectives	5	Specific objectives and hypotheses, *including the hypothesis concerning non-inferiority or equivalence*.	10
Outcomes	6	Clearly defined primary and secondary outcome measures *detailing whether the outcomes in the non-inferiority or equivalence trial are identical (or very similar) to those in any trial(s) that established efficacy of the reference treatment* and, when applicable, any methods used to enhance the quality of measurements (*e.g.*, multiple observations, training of assessors).	10
Sample size	7	How sample size was determined *detailing whether it was calculated using a non-inferiority or equivalence criterion and specifying the margin of equivalence with the rationale for its choice*. When applicable, explanation of any interim analyses and stopping rules (*and whether related to a non-inferiority or equivalence hypothesis*).	10,11
Randomization -- Sequence generation	8	Method used to generate the random allocation sequence, including details of any restrictions (*e.g.*, blocking, stratification)	8,9
Randomization -- Allocation concealment	9	Method used to implement the random allocation sequence (*e.g.*, numbered containers or central telephone), clarifying whether the sequence was concealed until interventions were assigned.	9
Randomization -- Implementation	10	Who generated the allocation sequence, who enrolled participants, and who assigned participants to their groups.	9
Blinding (masking)	11	Whether or not participants, those administering the interventions, and those assessing the outcomes were blinded to group assignment. If done, how the success of blinding was evaluated.	9
Statistical methods	12	Statistical methods used to compare groups for primary outcome(s), *specifying whether a one or two-sided confidence interval approach was used*. Methods for additional analyses, such as subgroup analyses and adjusted analyses.	11,12
RESULTS<br>Participant flow	13	Flow of participants through each stage (a diagram is strongly recommended). Specifically, for each group report the numbers of participants randomly assigned, receiving intended treatment, completing the study protocol, and analyzed for the primary outcome. Describe protocol deviations from study as planned, together with reasons.	12,13
Recruitment	14	Dates defining the periods of recruitment and follow-up.	12

Baseline data	15	Baseline demographic and clinical characteristics of each group.	Table 1
Numbers analyzed	16	Number of participants (denominator) in each group included in each analysis and whether the analysis was *"intention-to-treat" and/or alternative analyses were conducted*. State the results in absolute numbers when feasible (*e.g.*, 10/20, not 50%).	12
Outcomes and estimation	17	For each primary and secondary outcome, a summary of results for each group, and the estimated effect size and its precision (*e.g.*, 95% confidence interval). *For the outcome(s) for which non-inferiority or equivalence is hypothesized, a figure showing confidence intervals and margins of equivalence may be useful*.	Table 2
Ancillary analyses	18	Address multiplicity by reporting any other analyses performed, including subgroup analyses and adjusted analyses, indicating those pre-specified and those exploratory.	Table 3,4,5
Adverse events	19	All important adverse events or side effects in each intervention group.	Table 2
DISCUSSION Interpretation	20	Interpretation of the results, taking into account the *non-inferiority or equivalence hypothesis and any other* study hypotheses, sources of potential bias or imprecision and the dangers associated with multiplicity of analyses and outcomes.	16,17,18
Generalizability	21	Generalizability (external validity) of the trial findings.	19
Overall evidence	22	General interpretation of the results in the context of current evidence.	19,20

www.consort-statement.org
